# Exploring the Enigma: The Role of the Epithelial Protein Lost in Neoplasm in Normal Physiology and Cancer Pathogenesis

**DOI:** 10.3390/ijms25094970

**Published:** 2024-05-02

**Authors:** Emma Lindell, Xiaonan Zhang

**Affiliations:** Department of Immunology, Genetics and Pathology, Uppsala University, SE-751 85 Uppsala, Sweden; emma.lindell@igp.uu.se

**Keywords:** EPLIN/LIMA1, cancer progression, cytoskeleton dynamics, metabolism

## Abstract

The cytoskeleton plays a pivotal role in maintaining the epithelial phenotype and is vital to several hallmark processes of cancer. Over the past decades, researchers have identified the epithelial protein lost in neoplasm (EPLIN, also known as LIMA1) as a key regulator of cytoskeletal dynamics, cytoskeletal organization, motility, as well as cell growth and metabolism. Dysregulation of EPLIN is implicated in various aspects of cancer progression, such as tumor growth, invasion, metastasis, and therapeutic resistance. Its altered expression levels or activity can disrupt cytoskeletal dynamics, leading to aberrant cell motility and invasiveness characteristic of malignant cells. Moreover, the involvement of EPLIN in cell growth and metabolism underscores its significance in orchestrating key processes essential for cancer cell survival and proliferation. This review provides a comprehensive exploration of the intricate roles of EPLIN across diverse cellular processes in both normal physiology and cancer pathogenesis. Additionally, this review discusses the possibility of EPLIN as a potential target for anticancer therapy in future studies.

## 1. Introduction

The progression of tumors involves a complex series of events wherein cancer cells acquire mutations, enabling them to proliferate, invade surrounding tissues, and metastasize. This process is governed by various changes in both genotype and phenotype, collectively known as the hallmarks of cancer. These hallmarks include inducing angiogenesis, evading cell death, deregulating cellular metabolism, activating invasion and metastasis, sustaining proliferation, and inducing genome instability [1].

Throughout tumor progression, cancer cells demonstrate distinct gene expression profiles compared to normal cells. These alterations not only drive unchecked growth and metastasis but also present potential targets for precision medicine and avenues to differentiate between normal and cancerous cells [2]. The cytoskeleton, particularly the protein actin, is crucial for maintaining an epithelial phenotype and assumes a pivotal role in several hallmark processes of cancer. This includes upholding cell integrity, facilitating cell division, forming cell adhesions and junctions, promoting cell motility, and remodeling chromatin structure [3]. Metastatic cancer cells, characterized by their heightened motility and invasiveness, rely on dynamic actin cytoskeleton remodeling to migrate, invade surrounding tissues, and establish metastases in distant organs [4,5]. However, the precise cell signaling pathways governing cell–cell adhesion and actin cytoskeleton dynamics in metastatic cancer cells remain incompletely understood. 

In recent decades, researchers have identified the epithelial protein lost in neoplasm (EPLIN, also known as LIMA1) as a crucial component in regulating cytoskeletal dynamics (actin and β-catenin) and influencing alterations in cell motility and cell–cell adhesion [5,6]. In this review, we have provided an overview of EPLIN/LIMA1, discussing its fundamental characteristics and exploring its roles in various biological processes, tumorigenesis, and metastasis.

## 2. EPLIN (LIMA1)

**Figure 1:**
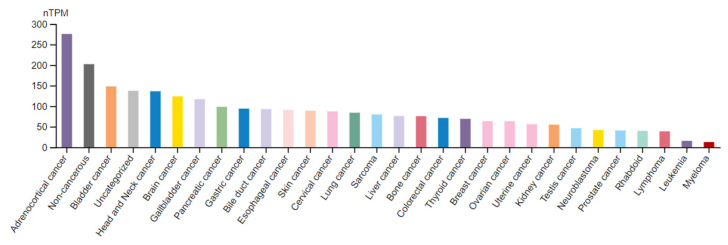
EPLIN is frequently subject to loss or downregulation in cancerous cells. RNA expression data are presented in the form of normalized transcript per million (nTPM) values for various cancer cell lines. These cell lines are categorized based on their respective cancer types. The figure was originally from the human protein atlas.

EPLIN was initially recognized as a novel cytoskeletal protein during a screening process, exhibiting preferential expression in human epithelial cells but often experiencing loss or downregulation in cancerous cells, where Maul and Chang first reported that the expression of EPLIN is downregulated in the majority of oral (8/8), prostate (7/7) and breast (5/6) cancer cells in 1999 [6]. However, it was not until a decade later that researcher delved into the expression profile of EPLIN in human cancer tissues. In their findings, the authors reported a decreased expression of EPLIN-α in breast tumor tissues. Additionally, patients with a poor prognosis exhibited significantly lower levels of EPLIN-α compared to those with a favorable prognosis [7]. In a separate study, researchers investigated the expression of EPLIN in a human bladder tissue microarray. They observed that EPLIN expression was reduced in cancerous tissues compared to its expression in normal tissues [8]. Similar findings have also been observed in gastric cancer, where EPLIN expression was associated with notable or nearly significant reductions in overall survival, disease-free survival, first-progression survival, or post-progression survival [9]. Steder et al. analyzed data from the international genomics consortium expression project for oncology and observed that the loss of EPLIN expression is strongly associated with the metastatic behavior and tumor grading of prostate, colon, and head and neck cancer [10]. We also examined the expression levels of EPLIN in various cancer cells using data from the human protein atlas. The findings revealed that the expression of EPLIN was generally reduced in most cancer cell types compared to non-cancerous cell lines (Figure 1).

**Figure 2:**
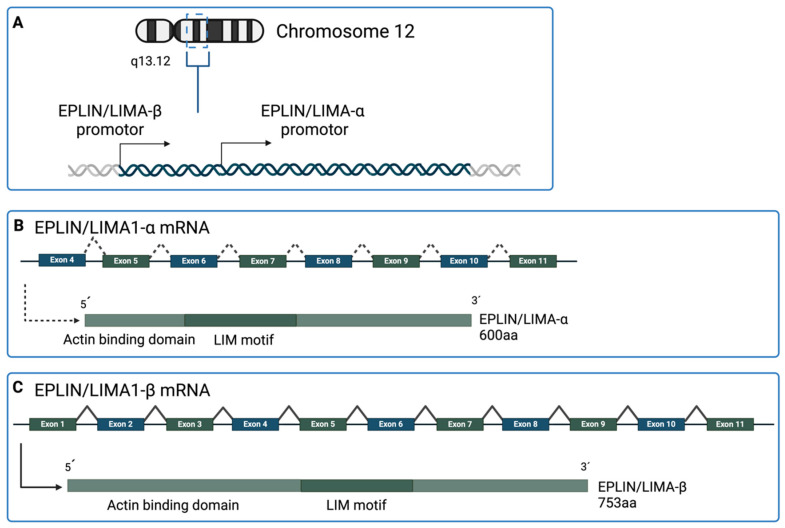
An illustration of the *EPLIN* gene. (**A**) *EPLIN* is located on chromosome 12 on the q13.12 position. *EPLIN* has two transcription start sites, resulting in the two isoforms EPLIN-α and EPLIN-β. (**B**) The *EPLIN-α* transcript contains exon 4 to exon 11 and includes 600aa. (**C**) *EPLIN-β* transcript includes all exons, from exon 1 to 11, and comprises 759aa. Both EPLIN isoforms include an actin-binding domain and a LIM domain, functioning as a centrally located interaction site. Created with BioRender.com (accessed on 30 January 2024).

*EPLIN* is situated on chromosome 12q13.12 with a sequence length of 107,733 bp, comprising 11 exons and 10 introns. The gene possesses two independent promoter regions, resulting in the production of two distinct isoforms: the smaller 600-residue EPLIN-α and the larger 759-residue EPLIN-β [6]. The transcription start site for EPLIN-β is situated proximal to exon 1, encompassing all 11 exons of the transcript. Conversely, the transcription start site for EPLIN-α is positioned approximately 50 kB downstream, preceding exon 4, and consequently encompasses exons 4–11 (Figure 2) [11]. Both EPLIN isoforms consist of two actin-binding domains (ABDs) and a central LIM domain. The LIM domain contains the transcription factors *Lin11*, *Isl-1*, and *Mec-4* (LIM). It includes a cysteine-rich (CX_2_-CX_16–23_-HX_2_-CX_2_-CX_2_-CX_16–21_-CX_2–3_-C/H/D) and a zinc-finger structural domain [12,13]. The LIM domain serves as a centrally located interaction site facilitating specific signaling transduction protein functions in EPLIN. This feature allows EPLIN to dimerize autonomously and to bind with other proteins [14]. So EPLIN was also known as the LIM domain and actin-binding protein 1 (LIMA1).

The isoforms of EPLIN, namely EPLIN-α and EPLIN-β, are conserved across species [15]. In subsequent studies by Maul et al., EPLIN was identified as homologous to mice and zebrafish [6]. In mice, EPLIN-α is composed of 593 amino acids (aa), whereas EPLIN-β comprises 753 aa. Both isoforms exhibit a notable correlation of 77% and 75% identity, respectively, to their human EPLIN counterparts. Genetic studies in zebrafish unveiled a single isoform of EPLIN, consisting of 692 aa. While there was no significant correlation observed between the EPLIN isoforms found in humans and zebrafish, with only 37% identity and 50% similarity, seven highly conserved regions were still identified [15].

The majority of the regulatory mechanisms underlying the differential expression of EPLIN-α and EPLIN-β have been undetermined since it was first reported in 1999 [6]. The promotor region for EPLIN-α contains a serum response factor (SRF) binding site, whilst the promotor for EPLIN-β includes a putative binding site for OCT-1, SP1, and AP1. In NIH3T3 fibroblast, serum stimulation or a temporary expression of several Rho-family small GTPases both stimulate SRF and promote transcription of EPLIN-α (the smaller isoform) but not in EPLIN-β, indicating that EPLIN-α is the main isoform in these cells [11].

## 3. Functions of EPLIN in Normal Cells

In healthy cells, EPLIN is vital in regulating fundamental cellular functions, including actin dynamics, cytoskeletal organization, motility, cell growth, and metabolism [8,11,14,16,17,18,19,20,21]. These functions are closely associated with its structure, comprising two actin-binding domains (ABDs) and a central LIM domain.

### 3.1. Role in Regulating Actin Dynamics, Cytoskeletal Organization, and Motility

The regulation of actin dynamics and cell motility by EPLIN is subject to various cellular signaling pathways. This includes the extracellular signal-regulated kinase (ERK) phosphorylation pathway, where EPLIN has been identified as a novel target of the ERK/MAPK signaling pathway. Phosphorylation of EPLIN by ERK can occur at residues Ser360, Ser602, and Ser692. This phosphorylation has been shown to increase cell motility while simultaneously reducing the affinity of actin filaments, as the binding activity at the C-terminal region is decreased [22], suggesting that ERK phosphorylation of EPLIN destabilizes the actin filaments. Furthermore, Chang et al. demonstrated that the regulation of EPLIN is influenced by human hCDC14A phosphatase (hCDC14A). hCDC14A dephosphorylates EPLIN at residues Ser362 and Ser604, leading to the bundling and stabilization of F-actin [23].

**Figure 3:**
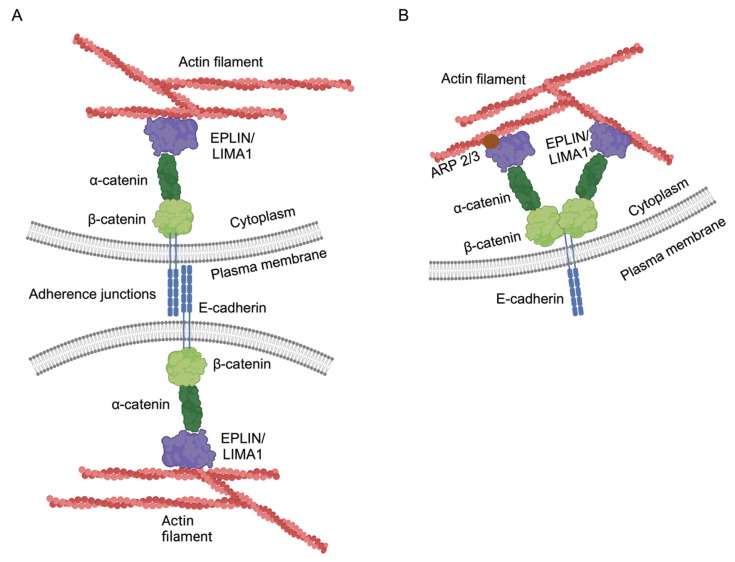
Schematic illustration of EPLIN and its association with adherens junctions. (**A**) EPLIN links F-actin and the cadherin–catenin complex as part of the adherens junction and is part of maintaining the apical–basal polarity of the epithelial cells. (**B**) EPLIN further interacts with additional proteins, such as ARP2/3 (brown dot), for stabilization of the actin filaments. Created with BioRender.com.

In epithelial cells, interactions between EPLIN and other proteins also play a crucial role in regulating actin dynamics, cytoskeletal organization, and motility. The actin filaments are connected to the cadherin–catenin complex through α-catenin at the adherence junctions (AJs) (Figure 3A) [24]. EPLIN plays a crucial role in mediating the interaction between the cadherin–catenin complex and F-actin at the apical cell–cell junctions. When EPLIN is depleted, it disrupts the assembly of apical actin and compromises the integrity of the adhesion belt. This suggests that EPLIN serves as a key component linking the cadherin–catenin complex to F-actin, thereby stabilizing both the adhesion belt and the cytoskeleton [21]. Actin filament assembly within cells necessitates the generation of new barbed ends, which can originate either de novo or through processes involving the removal of capping proteins or the severing of existing filaments, as described by Pollard et al. in 2000 [25]. One mechanism facilitating the creation of new barbed ends de novo involves the activation of the ARP2/3 (actin-related proteins 2/3) complex. EPLIN has been shown to inhibit the nucleation of actin filaments mediated by the ARP2/3 complex [14,21,26], leading to the establishment of cell polarity at the apical adhesion sites and the stabilization of the cytoskeleton (Figure 3B). EPLIN could also combine with vinculin, another protein essential for adherence junction formation [19]. EPLIN has moreover been demonstrated to interact with leucine zipper protein 1 (LUZP1). LUZP1 is localized to the actin filaments as well as the basal body, and, similar to EPLIN, it was discovered to take part in stabilizing the actin filaments and regulating their dynamics by assembling with ARP2. The depletion of EPLIN and LUZP1 further showed an increase in MyosinVa [26] another actin cytoskeleton-associated protein that promotes ciliary vesicle formation [27]. Recent studies have also demonstrated that EPLIN localizes to focal adhesions, where it interacts with paxillin, potentially contributing to the stabilization of focal adhesions [28], thus limiting cell motility.

### 3.2. Role in Regulating Cell Growth and Metabolism

In addition to its potential role in regulating actin dynamics, cytoskeletal organization, and motility, EPLIN has also been shown to play roles in cell growth and cellular metabolism.

*a.*  *Reduced levels of EPLIN facilitate cell growth*

The isoforms of EPLIN, namely EPLIN-α and EPLIN-β, were discovered in 1999 [6]. However, to date, the distinct roles of EPLIN-α and EPLIN-β remain largely unknown, and most research is still focused on the overall function of EPLIN rather than specifically investigating the roles of its isoforms. A finding discovered that the stable reduction of EPLIN could increase cell growth [29]. Furthermore, the expression of EPLIN has been demonstrated to suppress anchorage-independent growth [30]. Depletion of EPLIN using small-interfering RNA (siRNA) induces cytokinesis failure, indicating that the loss of EPLIN may lead to aneuploidy and contribute to genomic instability [20].

*b.*  *EPLIN regulates cholesterol absorption*

Cholesterol, a crucial lipid, plays a pivotal role in regulating membrane properties and modifying proteins such as Hedgehog and Smoothened [31,32,33]. It can be acquired through de novo biosynthesis and dietary absorption. NPC1L1 (NPC1-Like Intracellular Cholesterol Transporter 1) facilitates the transport of a significant amount of cholesterol to an intracellular cholesterol pool known as the endocytic recycling compartment [34]. When the cholesterol level in the endocytic recycling compartment decreases, NPC1L1 interacts with EPLIN through its Q1277KR residues. Subsequently, EPLIN, along with the associated myosin Vb, facilitates the recycling of NPC1L1 back to the plasma membrane [18]. Mutation of *EPLIN* leads to the intracellular retention of NPC1L1, resulting in decreased intestinal cholesterol absorption [18]. Consistent with these findings, another study by Xiao et al. demonstrated that *EPLIN* knockdown led to the entrapment of NPC1L1 in the endocytic recycling compartment rather than its return to the plasma membrane [16]. In another metabolomic, phenomics, and genomic study by Su et al., a number of genes were found to be associated with lipid metabolism. Notably, APOA5 showed a strong association, and HIF1A and EPLIN exhibited a lower association. The specific mechanism underlying the effect of EPLIN on lipid metabolism was not disclosed in the study, although the authors suggested that the effect may be connected to cholesterol absorption [35]. Collectively, these findings suggest that EPLIN may indeed play a role in regulating the level of cholesterol in cells.

*c.*  *EPLIN is essential for maintaining proper mitochondrial function*

In addition to affecting the level of cholesterol in cells, it has been suggested that EPLIN is necessary for proper mitochondrial function in a cell-autonomous manner. However, findings in this regard have been subject to debate and inconsistency. Studies conducted by the Fujita Y. group have indicated that the accumulation of EPLIN leads to a decrease in mitochondrial transmembrane potential (ΔΨm) through a non-cell-autonomous mechanism. Moreover, the knockdown of EPLIN in RasV12 cells has been shown to significantly restore mitochondrial membrane potential [36,37]. However, research by the Bedzhov group found that the depletion of EPLIN could result in a general reduction of the mitochondrial transmembrane potential, mitochondrial ATP production rate, and energy efficiency in a cell-autonomous manner [17]. The Bedzhov group also identified several potential interaction partners of EPLIN related to cellular metabolism, including pyruvate kinase and dihydropyrimidinase. Although none of these candidates directly control critical steps of energy homeostasis, it remains plausible that unidentified EPLIN interaction partner(s) may play a role in regulating mitochondrial metabolism involving pyruvate kinase. Therefore, a comprehensive understanding of how EPLIN influences mitochondrial function requires further investigation. Alternatively, the primary role of EPLIN as an actin-binding protein may indirectly impact cellular metabolism. For example, studies have demonstrated that the dynamics of the actin cytoskeleton regulate the activity of aldolase A, a pivotal glycolytic enzyme [38]. In addition, the application of force to the E-cadherin complex promotes the activity of AMP-activated protein kinase (AMPK), which plays a critical role in glucose and fatty acid uptake and oxidation [39].

## 4. The Diverse Role of EPLIN in Cancer

### 4.1. EPLIN Negatively Regulates Tumorigenesis

The role of EPLIN in tumorigenesis and metastasis remains a topic of debate. While some studies have indicated that elevated expression of EPLIN correlates with poor prognosis [40], the majority of studies still support the notion that EPLIN acts as a tumor suppressor across various malignancies. This is evident from a large number of findings showing that EPLIN is downregulated across various malignancies, including oral cancer [6], breast cancer [7,41], prostate cancer [42,43,44,45], lung cancer [15,46], and ovarian cancer [47]. The downregulation of EPLIN has been linked to poor prognosis in patients with these types of cancer.

*a.*  *EPLIN as a key defender in epithelial transformation*

During the early stages of cancer initiation, epithelial cells undergo a transformation triggered by mutations in oncoproteins. This transformation leads to the development of cancerous cells, which then compete with normal cells for survival within the tissue microenvironment. The survival of both normal and transformed cells is tightly regulated by mechanisms collectively known as the epithelial defense against cancer (EDAC). EDAC serves as the primary anti-tumor mechanism, acting as the first line of defense against cancer development and progression [48]. This phenomenon was initially discovered in Drosophila [49] and has subsequently been found to occur in mammals [48,50]. It has been demonstrated that in mammalian cell cultures, proto-oncogene tyrosine protein kinase (Src-), ErbB2-, or Ras-transformed cells are extruded from the epithelium [51,52]. The molecular mechanism behind this event, however, is not fully established.

In a study led by Ohoka et al., it was observed that both EPLIN and caveolin-1 (Cav-1) accumulated in RasV12-transformed cells, particularly in close proximity to normal cells. These proteins were identified in various cellular compartments, including the cytoplasmic matrix and the outer and apical membrane structural domains. When EPLIN or Cav-1 was knocked down, there was a notable suppression in the elimination of RasV12-transformed cells from the apical epithelium, indicating their involvement in removing transformed cells from the epithelium. Furthermore, EPLIN was found to regulate myosin II and protein kinase A (PKA) through non-cellular autonomous activation, primarily upstream of Cav-1 in RasV12-transformed cells. Additionally, EPLIN exerted significant effects on filamin, which is another crucial regulator involved in the epithelial defense against cancer (EDAC) [53].

In a subsequent study conducted by Saitoh et al., they delved deeper into the relationship between RasV12-transformed cells and EPLIN regulation within the context of EDAC. They uncovered that Rab5, an important regulator of endocytosis and membrane transport, orchestrated the endocytosis of E-cadherin and EPLIN in RasV12-transformed cells. Upon contact with normal cells, EPLIN relocated to the cytoplasm within RasV12-transformed cells [54]. Furthermore, EPLIN exhibited partial association with internalized E-cadherin and formed a complex that activated the PKA signaling pathway, facilitating the removal of cells from the epithelium. Notably, PKA activity was heightened in RasV12-transformed cells adjacent to normal cells, but co-expression of Rab5DN (a dominant-negative form of Rab5) significantly dampened this activity. Moreover, inhibition of PKA led to a marked suppression of the apical displacement of RasV12-transformed cells. Intriguingly, EPLIN knockdown did not impact Rab5 accumulation, suggesting that Rab5-regulated endocytosis operates upstream of the EPLIN/PKA pathway within the cellular context of EDAC [55].

In another investigation led by Kasai et al., paxillin emerged as a key regulator of the EPLIN/plectin complex, facilitating α-tubulin acetylation and subsequent microtubule reorganization. This complex, involving paxillin, plectin, and EPLIN, served as a crucial platform for the acetylation of tubulin via HDAC6. However, the inhibitory impact of paxillin knockdown on the apical extrusion of RasV12-transformed cells was only partially restored by suppressing HDAC6 activity. This suggests the involvement of additional molecules downstream of the paxillin–plectin–EPLIN complex, necessitating further exploration into the underlying mechanisms [56]. While paxillin knockdown rescued the apical extrusion of RasV12-transformed cells by suppressing HDAC6, it indicated the involvement of additional downstream molecules in the paxillin–plectin–EPLIN complex.

*b.*  *EPLIN, as a direct target of the p53 family, could interact and cooperate with p53*

P53 is a well-known tumor suppressor that functions as a transcription factor, playing a vast role in the regulation of the cell cycle, genome stability, and apoptosis [57]. Ohashi et al. conducted a ChIP-seq assay and identified peaks named EPLIN-RE1 and EPLIN-RE2 in the fourth intron and downstream region of the *EPLIN* gene, respectively. The nucleotide sequence of each peak matched the consensus *p53* motif (RRRCWWGYYY RRRCWWGYYY), suggesting that endogenous wild-type *p53* increased the induction of EPLIN protein expression. Additionally, they depleted EPLIN and treated cells with nutlin-3a, a small molecule activating endogenous p53, finding that the suppression of cancer cell invasion induced by p53 was impaired. This implies that the induction of EPLIN expression is necessary for the tumor suppressor functions of p53 [58]. In an independent investigation, Steder et al. discovered that DNp73, an inhibitor of the p53 tumor suppressor family, can decrease EPLIN expression. This leads to the disruption of adherens junctions and the metastatic potential in melanoma cells. The changes in cell adhesion and migration induced by DNp73 are mediated through the activation of AKT and STAT3 signaling pathways in response to EPLIN inhibition. Consequently, this results in the upregulation of SNAIL and simultaneous downregulation of E-cadherin. These findings suggest that EPLIN is a target of DNp73, and depleting EPLIN initiates the invasion-metastasis cascade [10].

*c.*  *EPLIN, identified as a binding target for MAD2, prevents tumor progression*

Abnormal expression of MAD2 (mitotic arrest deficient 2) has been linked to the initiation and advancement of various malignancies [59]. Additionally, the deubiquitinating enzyme USP44, a member of the cysteine protease family, acts as a crucial regulator of the spindle checkpoint, and patients with reduced USP44 levels exhibit poor overall survival and recurrence-free survival [60]. A published study has reported that in prostate cancer, the epidermal growth factor can stimulate the degradation of EPLIN through the ubiquitin–proteasome system [61]. In a study by Jiang et al., significant changes in the levels of EPLIN were observed in cells where MAD2 expression was manipulated. Specifically, EPLIN levels dramatically increased when MAD2 was downregulated, whereas they decreased upon MAD2 overexpression. Further investigation revealed that MAD2 expression levels influenced the binding affinity of USP44 to EPLIN. Higher MAD2 levels led to increased occupancy of USP44 in nuclei and EPLIN in the cytoplasm, resulting in reduced binding of USP44 to EPLIN and subsequent degradation of EPLIN at the protein level. Conversely, lower MAD2 levels had the opposite effect, promoting EPLIN stability. Additionally, the phosphorylation of PI3K and AKT was observed to be inactivated upon MAD2 knockdown but activated with MAD2 overexpression [62]. These findings collectively suggest that the MAD2-stimulated PI3K/AKT pathway is intricately regulated by the axis involving EPLIN. Increased levels of EPLIN could suppress MAD2, thereby potentially inhibiting tumor progression.

*d.*  *EPLIN negatively regulates the epithelial–mesenchymal transition event*

It is believed that the acquisition of migratory and invasive capabilities by cancer cells at the primary site marks the initial phase of tumor metastasis [63]. This process reflects epithelial–mesenchymal transition (EMT), a highly conserved cellular program observed during embryonic development. During EMT, epithelial cells lose their polarity and acquire motility by downregulating epithelial markers, disrupting the cadherin/catenin adhesion complex, and re-expressing mesenchymal molecules crucial for invasion and metastasis [64].

**Figure 4:**
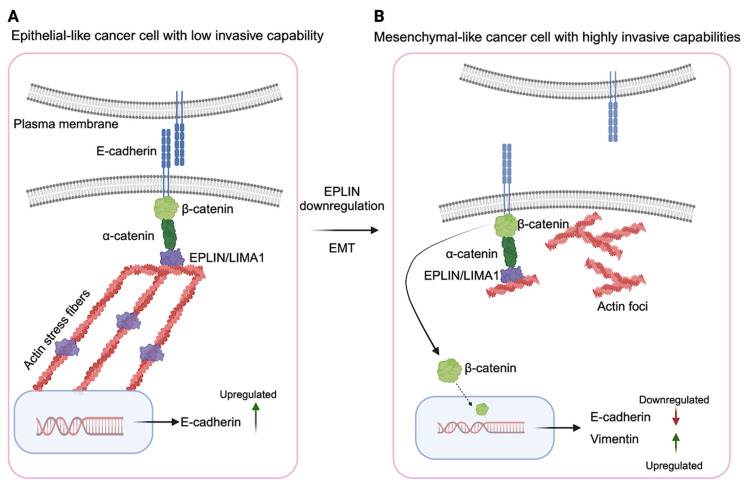
The representation of the role of EPLIN in epithelial–mesenchymal transition and metastasis. (**A**) EPLIN serves as a link between stress fibers (actin filaments) and the cadherin–catenin complex within these cells. (**B**) When EPLIN is downregulated, it leads to the dissociation of the adherens junction, remodeling of the actin cytoskeleton, and activation of the β-catenin signaling pathway. Consequently, this cascade of events results in the upregulation of EMT-promoting genes, such as Vimentin, and induces morphological changes that transform the cells into mesenchymal-like cancer cells with highly invasive capabilities. Created with BioRender.com.

The initiation of EMT marks a critical phase in cellular transformation, representing a dynamic process essential for embryonic development and implicated in tumor progression and metastasis in cancer. Various EMT-related factors and pathways, including SNAIL, WNT/β-catenin, and Hedgehog signaling, exhibit striking parallels between embryonic development and tumor progression [65,66]. EMT entails the dismantling of intercellular junctions crucial for maintaining epithelial integrity, including adherence junctions, tight junctions, gap junctions, and desmosomes, which undergo profound structural alterations. Notably, E-cadherins within adherence junctions undergo proteolytic cleavage and subsequent degradation, disrupting their association with β-catenin [64]. The progression of EMT is orchestrated by a complex interplay of signaling pathways that culminate in the activation of key transcription factors like SNAIL1 (or SLUG), which serve as master regulators driving the cellular transition from epithelial to mesenchymal states. Several signaling cascades, including the WNT/β-catenin, TGF-β, and Notch pathways, play pivotal roles in orchestrating EMT, exerting intricate control over cellular fate and phenotype. These pathways integrate diverse extracellular cues and intracellular signals to modulate gene expression, cytoskeletal dynamics, and cellular behavior, ultimately shaping the EMT process and impacting the invasive and metastatic potential of cancer cells (Figure 4) [64].

Zhang et al. conducted a study illustrating the role of EPLIN during EMT, highlighting its diminished expression as a characteristic feature. They observed that decreased EPLIN levels correlated with heightened invasiveness in prostate cancer cells, suggesting a negative regulatory role for EPLIN in impeding cancer cell invasion. Furthermore, their findings indicated that the knockdown of EPLIN led to the disruption of adherence junctions, prompting a restructuring of the actin cytoskeleton and activation of β-catenin signaling. These observations underscored the significance of EPLIN in maintaining epithelial integrity and restraining cellular motility. Immunohistochemistry staining of lymph node metastases from various solid cancers, including breast, prostate, colorectal, and squamous cell carcinoma, further revealed the downregulation of EPLIN [43].

In a subsequent investigation, Zhang et al. elucidated the role of epidermal growth factor, a known contributor to prostate cancer progression, in inducing EMT by negatively modulating EPLIN expression via ERK1/2 activation. Activation of the ERK1/2 signaling pathway resulted in the phosphorylation and subsequent degradation of EPLIN, leading to destabilization of actin filaments and disruption of adherence junctions, thus facilitating EMT progression [61]. Additionally, Steder et al. made a significant discovery regarding the role of DNp73 as a regulator of EPLIN expression in melanoma metastasis-induced EMT. They demonstrated that DNp73 suppressed both EPLIN-α and EPLIN-β expression, culminating in the activation of SNAIL1, loss of E-cadherin expression, and promotion of EMT [10].

### 4.2. EPLIN and Angiogenesis

Cancer cells induce angiogenesis, which is the process of generating new blood vessels from existing ones by endothelial cells. This phenomenon promotes the supply of oxygen and nutrients to cancer cells, enabling them to thrive and eliminating metabolic waste, similar to normal cells [67]. The initiation of angiogenesis involves activating the angiogenic switch, which leads to neovascularization. This intricate process entails the proliferation and migration of endothelial cells, ultimately resulting in the formation of new lumens and fully developed blood vessels [68]. VE-cadherins play a crucial role in maintaining vascular integrity by forming adherence junctions in vascular cells. These junctions are established through interactions between VE-cadherins and a variety of proteins, including β-catenin, p120, plakoglobin, α-catenin, α-actinin, N-WASP, and EPLIN. These interactions contribute to the structural stability and proper functioning of the endothelial barrier, ensuring the integrity of blood vessels [69]. For a significant duration, EPLIN has been recognized as a constituent of epithelial cells intimately associated with adherens junctions. However, Chervin-Petinot et al. expanded this understanding by uncovering the presence of both EPLIN-α and EPLIN-β mRNA and protein expression in endothelial cells, notably in human umbilical vein endothelial cells (HUVECs). Their investigation revealed a notable correspondence in the protein expression of EPLIN-α between epithelial and endothelial cells, whereas EPLIN-β exhibited comparatively lower expression levels in endothelial cells, likely influenced by differences in the activity of respective promoters. Moreover, they identified the localization of EPLIN to the plasma membrane, where it engaged with α-catenin and actin filaments and formed a complex with VE-cadherin. Consequently, they proposed a role for EPLIN in establishing cell–cell junctions in endothelial cells, aligning closely with their functional requirements. Their findings further underscored the indispensable nature of EPLIN in stabilizing capillary structures within an angiogenesis model [70]. In the study by Liang et al., it was observed that miR-93-5p downregulates EPLIN expression, which in turn enhances both growth and angiogenesis. These findings suggest that EPLIN may play an antagonistic role in angiogenesis [71].

### 4.3. EPLIN and Its Cancer-Promoting Role

While the majority of studies support the idea that EPLIN acts as a tumor suppressor across various malignancies, there are also reports suggesting a tumor-promoting role for EPLIN. For instance, EPLIN overexpression has been linked to EMT induction through the activation of cancer-promoting pathways like JAK/STAT and PI3K-AKT in head and neck squamous cell carcinoma [40]. In another study, EPLIN-β has been identified as a positive regulator of cellular migration, being upregulated and causal to enhanced cellular growth and migration in another study [72].

p62, recognized as a scaffold protein, participates in a multitude of cellular processes such as nutritional stress, oxidative stress, apoptosis, and autophagy. It is regarded as a carcinogenic protein, exhibiting high expression levels in tumors and influencing cell cycle regulation, apoptosis, invasion, and migration [73,74]. In a study conducted by Liu et al., the interaction between p62 and EPLIN was investigated [75]. The treatment of cells with the protein synthesis inhibitor cycloheximide (CHX) resulted in a faster reduction of p62 compared with controls, suggesting that p62 knockdown leads to faster degradation of EPLIN. GST pull-down experiments unveiled that p62 positively modulates EPLIN expression by increasing its protein stability within the cytoplasm. However, the relevant mechanism by which p62 affects EPLIN protein stability is still unclear.

These emerging findings present a seemingly contradictory scenario, highlighting the complex role of EPLIN across different stages and types of cancer, underscoring the necessity for further exploration and understanding of its intricate mechanisms in cancer biology.

### 4.4. EPLIN Exhibits Broad Interaction Spectra with Other Proteins within Cancer Cells

Since its initial report in 1999, EPLIN has not been a focal point of interest for scientists. However, recent studies have shed light on its involvement in facilitating extracellular matrix remodeling during cell migration, cellular metabolism, and its role in tumorigenesis and progression. As a result, EPLIN has garnered increased attention. To deepen our comprehension of its role in cellular functions, we have compiled a summary of its reported interaction activities with other proteins within cells, listed below (Table 1), suggesting that EPLIN may play a broader and undervalued role in cancer.

## 5. EPLIN in Different Cancer Types

Downregulation or loss of *EPLIN* has been observed in several solid tumors and has been linked to malignancies through various mechanisms. As a result, *EPLIN* is generally regarded as a tumor suppressor gene. However, as discussed earlier, the function of EPLIN can be contradictory in different cancer types or at different stages of cancer progression. To gain a better understanding of the role of EPLIN in tumors, it is important to discuss its role based on specific cancer types.

### 5.1. Hepatocellular Carcinoma

Hepatocellular carcinoma (HCC) is a prevalent liver cancer that often arises from cirrhosis or chronic liver diseases, posing significant mortality risks. Various molecular pathways contribute to HCC development, including WNT/β-catenin, TERT promoter mutations, Akt/mTOR, p53, VEGFR, and EGFR/RAS/MAPK pathways [76]. In a study led by Qi et al., EPLIN emerged as a pivotal tumor suppressor gene in hepatocellular carcinoma (HCC), marked by its downregulation within HCC tissues. The research uncovered a compelling association between reduced EPLIN expression and enhanced overall survival as well as recurrence-free survival among HCC patients. Notably, the study demonstrated that elevated EPLIN levels effectively suppressed HCC proliferation and metastasis, both in experimental models and clinical settings. In addition, they demonstrated that EPLIN functioned as an inhibitor of the Wnt/β-catenin signaling pathway through the interaction with BMI1 [76], a proto-oncogene contributing to the malignancy of HCC [77]. Additionally, Qi et al. delved into the role of miR-20a-5p, a small noncoding RNA previously implicated as an oncogene in breast and cervical cancer, in the context of HCC and its interplay with EPLIN expression. Their findings revealed that exosomes derived from cancer-associated fibroblasts enriched with miR-20a-5p effectively silenced EPLIN, thereby contributing to the suppression of HCC mediated by EPLIN [78].

### 5.2. Lymphomas

Mucosa-associated lymphoid tissue lymphoma (MALT lymphoma) accounts for approximately 8% of non-Hodgkin’s lymphomas and stands out as the most prevalent extranodal B-cell tumor. A characteristic chromosomal translocation observed in MALT lymphoma is t(11;18)(q21;q21), which gives rise to an apoptosis inhibitor 2-MALT lymphoma translocation gene 1 (API2-MALT1) fusion protein. Notably, MALT lymphomas harboring positive API2-MALT1 translocations exhibit aggressive behavior and demonstrate a higher proliferation rate compared to other subtypes [79]. Nie et al. made a significant discovery regarding the interaction between EPLIN-α and the API2-MALT1 protein, specifically in API2-MALT positive MALT lymphoma. They found that EPLIN-α interacts with the API2-MALT1 fusion protein but not with MALT1 alone, highlighting the necessity of the N-terminus replacement of MALT1 with API2 for this interaction. Moreover, their research revealed that API2-MALT1 paracaspase-mediated function leads to the cleavage of EPLIN-α, resulting in the loss of its tumor suppressor activity both in vitro and in vivo. Intriguingly, they also observed an oncogenic effect of the cleaved EPLIN-α residue, generating a LIM-domain-only protein, which further underscores the complex interplay between EPLIN-α and API2-MALT1 in MALT lymphoma progression [29].

### 5.3. Breast Cancer

Breast cancer stands as one of the foremost causes of cancer-related fatalities among women, notwithstanding the strides made in treatment advancements. It exhibits heterogeneity, manifesting distinct variations in molecular and pathological characteristics. Additionally, breast cancer is recognized as a hereditary disorder, often associated with mutations in the BRCA1/2 gene. Despite the advent of novel treatment modalities that have improved survival rates among breast cancer patients, mortality rates remain elevated, underscoring the imperative for continued research into molecular pathways and potential therapeutic targets in breast cancer treatment [80]. In breast cancer cells, in 1999, Maul et al. discovered the downregulation of EPLIN [6]. Jiang et al. conducted a study elucidating the significance of the EPLIN isoform EPLIN-α in breast cancer. Their findings revealed a correlation between the expression of EPLIN-α and patient outcomes, where lower levels of EPLIN-α were associated with good prognosis, highlighting its prognostic value. Elevated levels of EPLIN-α were observed to coincide with decreased proliferation rates of breast cancer cells, as evidenced in both in vitro and in vivo experiments. Moreover, the overexpression of EPLIN-α in MB-231 cells led to diminished migration and invasion capabilities, underscoring its potential role as a tumor suppressor in breast cancer progression [7]. In an additional study by Zhang et al., a relatively high expression of EPLIN was observed in breast cancer. However, the EPLIN expression was significantly lower in corresponding metastases to the lymph nodes [43].

### 5.4. Epithelial Ovarian Cancer

In a separate investigation focusing on ovarian cancer, researchers examined the expression of EPLIN-α, particularly emphasizing its relevance in processes like proliferation and neovascularization, which are critical in ovarian cancer progression. Their study revealed that EPLIN-α exhibited high expression levels in ovarian cancer cell lines SKOV3 and COV504. Remarkably, heightened expression of EPLIN-α was associated with the inhibition of aggressiveness in these cell lines. Upon knocking down EPLIN-α, an increase in proliferation was observed in vitro, underscoring its role in modulating cell growth dynamics. Furthermore, the knockdown of EPLIN-α led to alterations in cell matrix adhesions, compared to control cells expressing pEF6, suggesting its involvement in regulating cellular adhesion processes in ovarian cancer cells [47].

### 5.5. Head and Neck Squamous Cell Carcinoma

Head and neck squamous cell carcinoma (HNSCC) predominantly arises from the mucosal epithelium in regions such as the pharynx and larynx. It encompasses subtypes distinguished by their human papillomavirus (HPV) status, with HPV-positive variants prevalent in the pharynx and HPV-negative types more frequently found in the larynx and oral cavity [81]. Ma et al. conducted a comprehensive study examining the expression of EPLIN in HNSCC, revealing significant associations with disease progression. Their findings suggested that EPLIN overexpression was particularly pronounced in HPV-negative HNSCC cases with TP53 mutations, indicating its potential role as an oncogene in HNSCC progression and its possible utility as a biomarker for this specific subtype. Pathway analysis conducted in the study revealed enrichment of PI3-AKT and JAK/STAT pathways in HNSCC gene sets containing EPLIN, further highlighting the aggressive role of EPLIN in the development of HNSCC [40]. Furthermore, EPLIN was assessed as a potential prognostic biomarker in a cohort of oral tongue cancer patients comprising 120 individuals by Wirsing et al. However, their analysis did not reveal significant prognostic value associated with EPLIN in this particular cohort [82].

### 5.6. Cancers of Digestive System (Esophageal and Gastric)

Gastric cancer has become increasingly prevalent and poses a significant health challenge due to its aggressive nature and late-stage diagnosis [83]. Research examining the expression of EPLIN in clinical cohorts of gastric cancer has unveiled its potential as a prognostic biomarker and its correlation with the response to neoadjuvant chemotherapy (NAC). Low levels of EPLIN expression have been associated with increased infiltration and diminished tumor differentiation in patients with gastric cancer. Moreover, in these cohorts, EPLIN has been identified as an independent prognostic indicator for both overall survival and disease-free survival [9].

Esophageal cancer encompasses two primary types: esophageal squamous cell carcinoma (OSCC), sharing characteristics with head and neck squamous cell carcinoma (HNSCC), and adenocarcinoma (OAC), whose incidence is increasing in developed nations and shares genetic instability features with gastric cancer [84]. In a study conducted by Liu et al., the expression of EPLIN-α was assessed in esophageal cancer cells. They noted lower levels of EPLIN-α expression in esophageal cancer tissues compared to normal cells. Furthermore, the overexpression of EPLIN-α in the esophageal cancer cell line KYSE150 led to reduced cell aggressiveness, as evidenced by inhibited invasion [85]. These findings shed light on the potential role of EPLIN-α as a therapeutic target in managing esophageal cancer progression.

### 5.7. Prostate Cancer

Prostate cancer, known for its heterogeneity and complexity, poses a substantial health risk among men, spanning from slow-growing forms to aggressive, life-threatening tumors [86]. Diagnosing and treating prostate cancer present challenges due to the lack of specificity, often resulting in overtreatment and overdiagnosis. This underscores the urgent need for novel diagnostic biomarkers and therapeutic targets to enable precision cancer treatment strategies.

Early investigations by Maul et al. in 1999 identified the downregulation of EPLIN-α in prostate cancer, mirroring observations in breast and oral cancer cell lines, suggesting its role as a tumor suppressor gene. Subsequent studies by Zhang et al. revealed that EPLIN is highly expressed in epithelial prostate cancer cells with low invasiveness, contrasting with decreased expression in highly invasive prostate cancer cells. Depletion of EPLIN in prostate cancer cells led to the disassembly of apical adherence junctions, potentially slowing the cell cycle and suppressing proliferation. Furthermore, EPLIN depletion affected the expression of genes associated with epithelial–mesenchymal transition, including ZEB1, IGFBP-1, matrix metalloproteinases, and versican [43]. In aggressive PC-3 prostate cancer cell lines, Sanders et al. investigated the role of EPLIN-α in a cohort of prostate tissues. They found that EPLIN-α overexpression reduced the growth rate, inhibited the invasive effects of HGF cytokines, and influenced cell matrix adhesions in vitro. In vivo studies with EPLIN-α overexpression in PC-3 cells injected into mice demonstrated reduced tumor development [42]. Collins et al. similarly observed that EPLIN-α overexpression in prostate cancer cells led to reduced migration, invasion, and proliferation. They also noted transcriptional changes in paxillin, tyrosine protein kinase, and focal adhesion kinase, providing insights into the mechanisms underlying prostate cancer cell behavior [44]. Furthermore, Zhang et al. demonstrated that EGF, acting through the ERK1/2 pathway, induced the degradation of EPLIN, subsequently impacting the invasiveness of prostate cancer cells and epithelial–mesenchymal transition [61].

In summary, we comprehensively reviewed the involvement of EPLIN across different cancer types and its multifaceted roles in tumor biology in Table 2.

## 6. EPLIN as a Target in Cancer Therapies

The absence of EPLIN can disrupt cytoskeletal dynamics, impede cell motility, and compromise cell–cell adhesion, all of which are often linked to the facilitation of tumor proliferation, invasion, and migration. Hence, targeting EPLIN has emerged as a promising therapeutic avenue.

In a study exploring EMT in prostate cancer, investigators noted a pronounced decrease in EPLIN levels during EMT. Interestingly, they observed that depleting EPLIN notably enhanced cell resistance to docetaxel and doxorubicin treatment [43]. In a comprehensive clinical study focusing on gastric cancer, Gong et al. investigated the expression patterns of EPLIN transcripts alongside various clinicopathological parameters and responses to neoadjuvant chemotherapy across two distinct cohorts. Their analysis revealed a significant correlation between reduced EPLIN expression and decreased overall, disease-free, first-progression, or post-progression survival rates, particularly notable in the larger cohort. Additionally, they observed markedly higher levels of EPLIN expression in the combined T1 + T2 gastric cancer group compared to the T3 + T4 group, indicating a potential association with tumor staging. Furthermore, through multivariate analysis, they identified EPLIN expression as an independent prognostic factor for both disease-free survival and overall survival in the larger cohort [9]. These findings highlight the prognostic value of EPLIN expression in cancers and its potential as a predictive biomarker or target for chemotherapy.

The association between elevated serum cholesterol levels and heightened cancer risk, spanning various types such as colon, rectal, prostatic, and testicular cancer, highlights the significance of cholesterol metabolism in cancer progression and treatment response [87]. Recent studies have indicated that adjusting EPLIN levels might provide a sophisticated means of interfering with cholesterol-related pathways [18,88,89]. By delving into the mechanisms through which EPLIN governs cholesterol metabolism, novel therapeutic targets and strategies could be unearthed, capitalizing on the metabolic weaknesses inherent in cancer cells.

In the realm of tumor biology, membrane blebbing emerges as a significant mechanism facilitating cell motility, providing malignant cells with an alternative route for migration that diverges from the conventional reliance on extracellular matrix degradation [90]. This distinctive blebbing motility attribute grants resistance against anticancer treatments relying on pharmacological protease inhibitors [91]. Particularly noteworthy, Duethorn et al. observed that EPLIN depletion in pluripotent cells exacerbates blebbing [17], suggesting that in pathological conditions like cancer, this phenomenon may amplify tumor resistance to protease inhibitors. Furthermore, EPLIN has been noted to interact and synergize with p53, a renowned tumor suppressor [57]. Studies, including that of Ohashi et al., propose that inducing EPLIN expression is crucial for the tumor suppressor functions of p53 [58]. Consequently, strategies aimed at stabilizing EPLIN during early cancer stages or reinstating its function during metastasis could serve as pivotal therapeutic avenues for cancer treatments involving protease inhibitors alongside investigations centered on p53-related mechanisms.

## 7. Conclusions

Although EPLIN is increasingly recognized for its diverse roles in cellular processes, it remains a novel and mysterious molecule in the field of cancer biology. Its multifaceted involvement in key cellular processes such as motility, invasion, metastasis, lipid metabolism, and mitochondrial function underscores its complexity and importance in cancer progression. Our understanding of the complex functions and regulatory mechanisms of EPLIN, including its precise molecular interactions, upstream regulators, downstream effectors, and tissue-specific functions, remains incomplete. Therefore, EPLIN and its subtle role in cancer biology are critical to uncovering its full therapeutic potential and advancing the development of targeted anticancer strategies. These efforts have the potential to revolutionize cancer treatment paradigms, bring new hope to patients facing various cancer types, and improve their overall outcomes and quality of life.

## Figures and Tables

**Table 1:** EPLIN interacts with various “regulators” and plays a significant functional role in cellular processes.

EPLIN-Associated “Regulators”	Functional Role with EPLIN	References
ERK1/2	Regulates EPLIN through phosphorylation, increasing cell motility, and reduces the affinity of actin filaments, resulting in destabilization of actin filaments.	[22]
hCDC14A	Dephosphorylates EPLIN, resulting in stabilization of F-actin.	[23]
Cadherin–catenin, F-actin	Mediates interaction between cadherin–catenin and the F-actin complex, stabilizing the cytoskeleton.	[21]
ARP2/3	Eplin stabilizes cytoskeleton by its association with ARP2/3 by inhibiting nucleation of actin filaments.	[14,21]
Vinculin	Maintains the zonula adherens.	[19]
LUZP1	Regulating the actin dynamics and ciliary vesicle formation in association with Myosin Va.	[26,27]
NPC1L1	Interacts with EPLIN at low cholesterol levels facilitating the intracellular retention of NPC1L1.	[18]
Myosin II, PKA, and Cav-1	EPLIN is associated with Myosin II and PKA upstream of Cav-1 in the cytoplasmic matrix and apical membrane structure domains in the removal of transformed cells from the epithelium as a regulator of EDAC.	[53]
Rab-5	Regulator of endocytosis of E-cadherin and EPLIN in RasV12-transformed cells.	[54]
Paxillin, Plectin	Paxillin and plectin form a complex with EPLIN in RasV12-transformed cells regulating the apical extrusion.	[56]
p53	P53 increases the induction of EPLIN protein expression. The depletion of EPLIN suppresses the cancer cell invasion induced by p53.	[58]
DNp73 AKT/STAT-3, SNAIL	Has the ability to downregulate EPLIN, leading to the disruption of AJ. DNp73 and EPLIN interaction induces the phosphorylation of AKT/STAT-3 and, therefore, upregulation of SNAIL and the downregulation of E-cadherin.	[10]
MAD2, USP44	EPLIN levels are increased in MAD2 downregulated cells, and the MAD2 expression is associated with the interaction between USP44 and EPLIN.	[10,62]
JAK/STAT, PI3K-AKT	Associated with EMT induction as a result of EPLIN overexpression.	[40]
p62	Enhances the stability of EPLIN in the cytoplasm.	[75]
miR-93-5p	Downregulates EPLIN expression, enhancing growth and angiogenesis in HUVECs.	[71]

**Table 2:** Expression and functions of EPLIN in different cancer types.

Cancer Type	EPLIN Event	Functions	References
Hepatocellular carcinoma (HCC)	Downregulated in HCC tumor tissues	Correlated to decreased overall survival	[78]
	Overexpressed	Suppressed proliferation and metastasis	[78]
Lymphomas	Cleaved EPLIN-α	Loose tumor suppressor role and obtains oncogenic effect	[29,79]
Breast cancer	Overexpressed	Decreased proliferation, migration, and invasion	[7]
Epithelial ovarian cancer	Upregulated	Inhibition of aggressiveness	[47]
	Knocked down	Increased proliferation Regulation of adhesion process	[47]
Head and neck squamous carcinoma (HNSCC)	Overexpression (prominent in HPV-negative HNSCC)	Oncogene, associated with cancer progression.	[40]
Gastric cancer	Downregulated	Increased infiltration and reduced tumor differentiation Correlated to NAC chemosensitivity	[9]
Esophageal cancer	Overexpression	Reduced invasion and proliferation	[85]
Prostate cancer	Overexpression in tumor tissue	Reduced invasiveness	[43]
		Reduced growth rate and inhibited invasiveness. Reduced tumor development in vivo.	[42]
		Reduced migration, invasion, and proliferation.	[44]
	Depletion	Decreased cell cycle and suppressed proliferation	[43]
	Degradation of EPLIN	Reduced invasiveness.	[61]

## Data Availability

Not applicable.

## Abbreviations

EPLIN	Epithelial protein lost in neoplasm
LIMA1	LIM domain and actin-binding 1
LIM	Lin-11, Isl-1, and Mec-4
LUZP1	Leucine zipper protein1
ARP2	Actin-related protein
NPC1L1	NPC1-Like Intracellular Cholesterol Transporter 1
APOA5	Apolipoprotein A-5
ΔΨm	Mitochondrial transmembrane potential
AMPK	AMP-activated protein kinase
EDAC	Epithelial defense against cancer
Src	Proto-oncogene tyrosine protein kinase
Cav-1	Caveolin-1
PKA	Protein kinase A
EDAC	Epithelial defense against cancer
HDAC6	Histone deacetylates 6
MAD2	Mitotic arrest deficient 2
EMT	Epithelial–mesenchymal transition
HUVECs	Human umbilical vein endothelial cells
HCC	Hepatocellular carcinoma
TERT	Telomerase reverse transcriptase
MALT-lymphoma	Mucosa-associated lymphoid tissue lymphoma
API2	Apoptosis inhibitor 2
API2-MALT1	Apoptosis inhibitor 2-MALT lymphoma translocation gene 1
HNSCC	Head and neck squamous cell carcinoma
NAC	Neoadjuvant chemotherapy
OSCC	Esophageal squamous cell carcinoma
OAC	Adenocarcinoma
